# Retraction: Use of Granulocyte Colony-Stimulating Factor for the Treatment of Thin Endometrium in Experimental Rats

**DOI:** 10.1371/journal.pone.0228462

**Published:** 2020-01-24

**Authors:** The PLOS ONE Editors

After this article [1] was published, *PLOS ONE* was notified of similarities between lanes 1–3 in the Vimentin, CK19, and β-actin blots shown in Fig 3 of the *PLOS ONE* article, and panels in Fig 3A of a *Fertility and Sterility* article [2]. The western blot data represented results of different experiments in the two articles as detailed in the Methods sections. [1] and [2] were under review during overlapping periods, but the authors did not declare the related submission to the *PLOS ONE* Editors as is required by journal policy.

Lanes 1–3 of the Vimentin and β-actin panels and lane 1, 4 of the CK19 panel in Fig 3 [1] are also similar to results reported in Fig 3A of [3] (later corrected in [4] to provide updated Figs 2–4). The figures in these articles were used to represent results of different experiments, as detailed in the Methods sections.

The authors commented that they had made an error in preparing Fig 3 for the *PLOS ONE* article, and provided images in support of the CK19 and Vimentin results. However, these files did not clarify the concerns about data reporting in Fig 3. The authors noted that the western blots were done by a colleague whose contributions were not indicated in the published article, and that the authors received the results in figure form but did not obtain the original raw blot images. The underlying data for the western blots and for other results reported in the article are not available.

The authors apologized for the above issues and requested retraction of the *PLOS ONE* article, yet also commented that they are confident in the findings and stand by the published results.

In light of the above concerns, the *PLOS ONE* Editors retract this article.

Note, Fig 3 [1] reports material from [2], published earlier in 2013 [Elsevier, Inc. on behalf of the American Society for Reproductive Medicine], which is not offered under a CC-BY license. Fig 3 is therefore excluded from this article’s [1] license. At the time of retraction, the article [1] was republished to note this exclusion in the Fig 3 legend and the article’s copyright statement.

JZ, TT, YL agreed with retraction. QZ, YW either could not be reached or did not respond directly.
